# Dual-Functional Drug Delivery System for Bisphosphonate-Related Osteonecrosis Prevention and Its Bioinspired Releasing Model and In Vitro Assessment

**DOI:** 10.1021/acsomega.3c03440

**Published:** 2023-07-14

**Authors:** Piyarat Sungkhaphan, Boonlom Thavornyutikarn, Papon Muangsanit, Pakkanun Kaewkong, Setthawut Kitpakornsanti, Soraya Pornsuwan, Weerachai Singhatanadgit, Wanida Janvikul

**Affiliations:** † National Metal and Materials Technology Center, National Science and Technology Development Agency, Khlong Luang 12120, Thailand; ‡ National Center for Genetic Engineering and Biotechnology, National Science and Technology Development Agency, Khlong Luang 12120, Thailand; § Faculty of Dentistry and Research Unit in Mineralized Tissue Reconstruction, Thammasat University (Rangsit Campus), Khlong Luang 12120, Thailand; ∥ Faculty of Science, Mahidol University, Bangkok 10400, Thailand

## Abstract

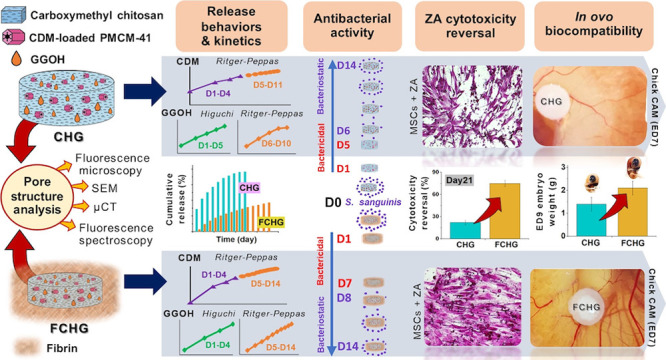

Clindamycin (CDM)/geranylgeraniol (GGOH)-loaded plasma-treated mesoporous silica nanoparticles/carboxymethyl chitosan composite hydrogels (CHG60 and CHG120) were developed for the prevention of medication-related osteonecrosis of the jaw associated with bisphosphonates (MRONJ-B). The pore structure and performances of CHGs, e.g., drug release profiles and kinetics, antibacterial activity, zoledronic acid (ZA)-induced cytotoxicity reversal activity, and acute cytotoxicity, were evaluated. The bioinspired platform mimicking in vivo fibrin matrices was also proposed for the in vitro/in vivo correlation. CHG120 was further encapsulated in the human-derived fibrin, generating FCHG120. The SEM and μCT images revealed the interconnected porous structures of CHG120 in both pure and fibrin-surrounding hydrogels with %porosity of 75 and 36%, respectively, indicating the presence of fibrin inside the hydrogel pores, besides its peripheral region, which was evidenced by confocal microscopy. The co-presence of GGOH moderately decelerated the overall releases of CDM from CHGs in the studied releasing fluids, i.e., phosphate buffer saline-based fluid (PBB) and simulated interstitial fluid (SIF). The whole-lifetime release patterns of CDM, fitted by the Ritger–Peppas equation, appeared nondifferentiable, divided into two releasing stages, i.e., rapid and steady releasing stages, whereas the biphasic drug release patterns of GGOH were observed with Phase I and II releases fitted by the Higuchi and Ritger–Peppas equations, respectively. Notably, the burst releases of both drugs were subsided with lengthier durations (up to 10–12 days) in SIF, compared with those in PBB, enabling CHGs to elicit satisfactory antibacterial and ZA cytotoxicity reversal activities for MRONJ-B prevention. The fibrin network in FCHG120 further reduced and sustained the drug releases for at least 14 days, lengthening bactericidal and ZA cytotoxicity reversal activities of FCHG and decreasing in vitro and in ovo acute drug toxicity. This highlighted the significance of fibrin matrices as appropriate in vivo-like platforms to evaluate the performance of an implant.

## 1 Introduction

Medication-related osteonecrosis of the jaw (MRONJ) associated with bisphosphonates is a serious adverse effect of long-term use of drugs, such as zoledronic acid (ZA) that is an antiresorptive drug commonly used in osteoporosis and oncology patients.^1,2^ MRONJ causes pain, a necrotic jawbone, and bacterial infection.^3^ Currently, no clinical practice guidelines are available for the management of MRONJ; therefore, standard supportive care is recommended.^2^ Preventive measures to reduce the risk of developing MRONJ remain challenging.

The potential cytotoxicity of ZA, which decreases the number of viable mesenchymal stem cells (MSCs), and thus functionally active osteoblasts, can induce bone necrosis after a jawbone injury.^4^ Infection with viridans streptococci is also associated with an increased risk of developing MRONJ.^3,5^ We have previously shown that geranylgeraniol (GGOH) could reverse ZA cytotoxicity at cellular and molecular levels.^6^ Clindamycin (CDM), a highly active antibiotic against viridans streptococci and also against bone infection,^7^ has been successfully incorporated in the biocompatible carriers.^8^ The developed CDM-loaded mesoporous silica nanoparticles/carboxymethyl chitosan composite hydrogels possessed an antibacterial activity and an osteogenic-inducing activity. However, a single drug delivery system often could not fulfill the needs of multiple-purpose clinical therapy. It is thus possible that a biodegradable and biocompatible carrier with sustained deliveries of dual drugs, i.e., CDM and GGOH, may be beneficial for the prevention of infected necrotic jawbone in MRONJ.

Several attempts have been made to develop dual drug delivery systems, particularly those based on hydrogels integrated with nanoparticles. Mobil Composition of Matter No.41 (MCM-41), a mesoporous material with an ordering hexagonal arrangement of cylindrical mesopores, has great potential in drug delivery applications owing to its high specific surface area and porosity, allowing drugs to be highly adsorbed inside the nanopores, as well as the outer surface of MCM-41.^9^ Biopolymers, e.g., chitosan, carboxymethyl chitosan, sodium hyaluronate, and gelatin, have been widely used as hydrogels for controlled drug delivery due to their excellent biocompatibility, biodegradability, and ease of gel-formation.^10−12^ The combinatorial uses of MCM-41 and biopolymers have attracted increasing attention for dual drug delivery systems.^10,12^ The resulting dual drug-loaded composite hydrogels explicitly exhibited broadened applications or greater performances compared to those of single drug-loaded hydrogels.^10^

Following the implantation of a drug-loaded carrier, the first and unavoidable event in tissue response is its direct contact with blood, leading to a blood clot formation surrounding (and, in some cases, within) the implanted material. Blood clot helps initiate hemostasis and facilitates cellular activities and a new extracellular matrix deposition. Fibrin, one of the most important proteins in human plasma, forms a natural nano-sized three-dimensional matrix in a blood clot. The presence of a fibrin matrix and interstitial body fluid at the implanted site may determine the in vivo levels of drug concentration in the tissue surrounding the implanted material by resisting drug liberation and thus altering the release kinetics. However, this important role of a fibrin-containing matrix in the kinetics of drug releases and supporting healing is often virtually ignored, leading to poor in vitro/in vivo correlations.

Herein, we report for the first time, the development of a dual-functional drug delivery system using a composite matrix platform to prevent MRONJ associated with bisphosphonates. The composite hydrogel, comprising carboxymethyl chitosan and plasma-treated MCM-41 nanoparticles, was co-loaded with CDM and GGOH. The resulting dual drug-loaded hydrogel was subsequently embedded in a human-derived fibrin gel network to mimic the in vivo implantation of the drug-loaded carrier that is supposed to be surrounded by a fibrin matrix found in a human blood clot. We comparatively examined how the morphology and internal structure of the dual drug-loaded composite hydrogels prepared with and without the fibrin gel network affected the release behaviors and kinetics of both drugs, as well as the biological properties, in terms of antibacterial activity, (acute) toxicity, and ZA reversal potency of the materials. In addition, the effects of amounts of drugs loaded in the composite carriers and types of drug-releasing media used on the performances of the materials were simultaneously assessed.

## 2 Materials and Experiments

### 2.1 Materials

Water-soluble carboxymethyl chitosan (coded as CM) (*M̅*_w_ = 3.0 × 10^5^ Da, degree of substitution (DS) = 0.9) and MCM-41 (alternately coded as M) mesoporous silica nanomaterial were directly prepared in our laboratory, according to the method described in the literature.^13^ Clindamycin hydrochloride (CDM, alternately coded as C) (MW = 479.46 g/mol), geranylgeraniol (GGOH, alternately coded as G) (MW = 290.48 g/mol), fibrinogen from human plasma (F3879), aprotinin from bovine lung (A1153), and thrombin from human plasma (T1063) were supplied by Sigma-Aldrich Corporation. Absolute ethanol (EtOH), methanol (MeOH) and acetonitrile (HPLC grade, 99.9%) were purchased from CT Chemical Co., Ltd. (Thailand). All analytical-grade chemicals were used as received without further purification.

### 2.2 Preparation of Drug-Loaded Mesoporous Silica Nanoparticles

Plasma-treated MCM-41 nanoparticles (encoded as both PMCM-41 and PM) were prepared, according to our previous study,^8^ via a low-pressure oxygen plasma treatment of MCM-41 for 1 h using inductively coupled RF-plasma (13.56 MHz). The essential properties of PMCM-41 were thoroughly analyzed and reported in the literature, i.e., phase composition by X-ray diffraction (XRD), specific surface area and pore characteristics by Brunauer–Emmett–Teller (BET), and elemental composition and surface chemical property by X-ray photoelectron spectroscopy (XPS). Afterward, CDM-loaded PMCM-41 (denoted as PMC) was formed using a conventional adsorption method by impregnating 420 mg of PMCM-41 in the solution of 200 mg of CDM in 3 mL of de-ionized (DI) water at room temperature for 24 h.^8^

### 2.3 Preparation of Dual Drug-Loaded Composite Hydrogels

**Scheme 1:**
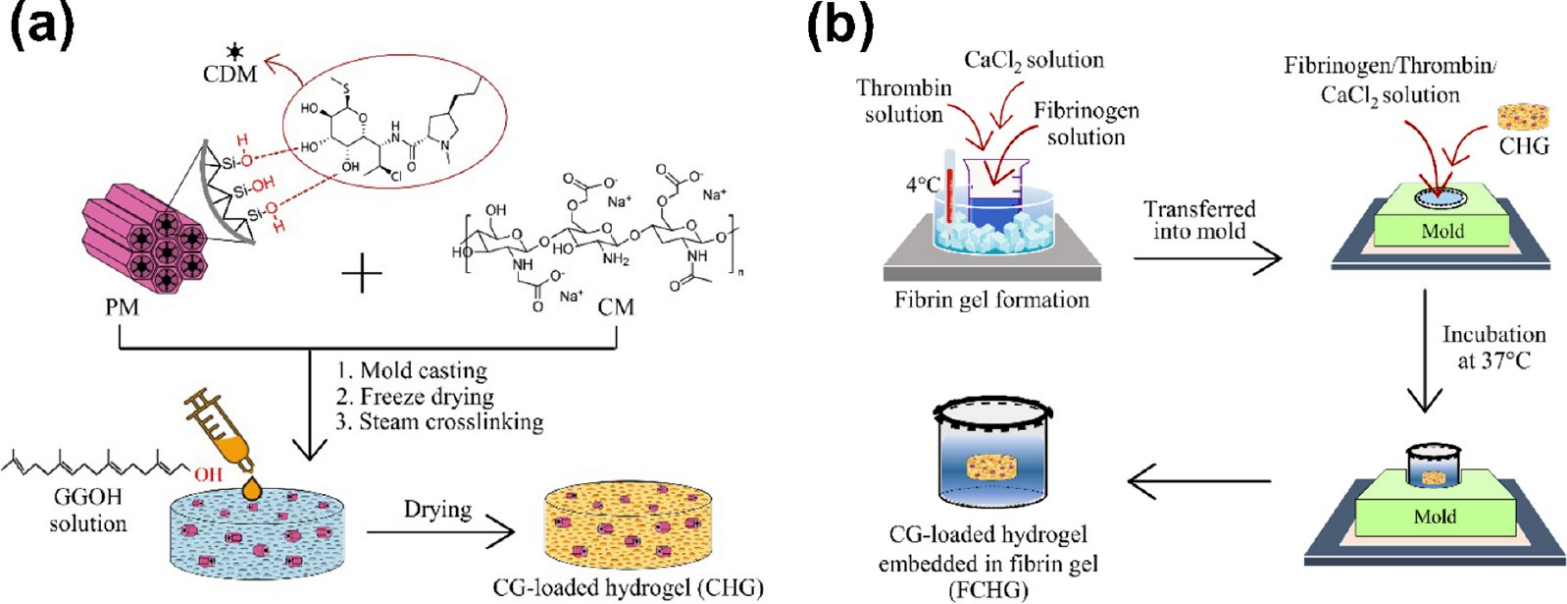
Schematic Illustration of Preparations of CG-loaded Composite Hydrogel (CHG) (a) and CG-loaded Hydrogel Embedded in Fibrin Gel (FCHG) (b)

In this study, two model drugs with different hydrophilicity were used: CDM, a water-soluble antibiotic medication, and GGOH, a poorly water-soluble monoterpenoid alcohol. The CG-loaded composite hydrogel (encoded as CHG) was prepared through a sequential process. CDM-loaded composite hydrogel (coded as CH) was first prepared, followed by the loading of GGOH onto the CH specimens. In the former step, as fully described in our previous report,^8^ the lyophilized CDM-loaded sponge-like pad which was primarily composed of CM and PMC (prepared in Section 2.2 above) mixed at a fixed weight ratio of 60: 40, was exposed to hot steam at 105 °C for 10 min to gently crosslink the polymer matrix, dried in a vacuum oven, and ultimately cut into disks (4 mm diameter × 2 mm thickness). In the latter step, 20 or 40 μL of 10 mM GGOH in EtOH, equivalent to 60 or 120 μg of GGOH, respectively, was loaded onto a CH disk by dropping half of the drug solution on the surface of one side of the disk and then air drying the whole disk for 1 h before repeating the same procedure for loading the remaining drug solution on the surface of the other side of the disk. Finally, the entire specimen was vacuum oven-dried for 3 h. The entire preparation process was schematically depicted in Scheme 1a. Two different dual drug-loaded composite hydrogels were generated in this study, namely, CHG60 and CHG120, whose numbers were encrypted based on the amounts (μg) of GGOH loaded.

### 2.4 Preparation of CHG-Embedded Fibrin Gel

Fibrinogen was reconstituted in prewarmed 0.9% normal saline mixed with 1500 KIU/mL of aprotinin to achieve a final concentration of 20 mg/mL. Thrombin was reconstituted in 40 mM of CaCl_2_ at 100 IU/mL. Typically, to prepare a fibrin gel (coded as F), 125 μL of 20 mg/mL fibrinogen was sequentially pre-mixed with 330 μL of Milli-Q water, 5 μL of 1 M CaCl_2_, and 40 μL of 100 IU/mL thrombin at 4 °C. Thus, the final concentration of fibrinogen used in the reaction was 5 mg/mL, which is normally found in human plasma.^14^ To prepare the CG-loaded composite hydrogel embedded in a fibrin gel, 250 μL of the pre-mixed fibrinogen/thrombin/CaCl_2_ solution was first pipetted into an open-ended Eppendorf tube (10 mm diameter × 12 mm height) inserted in a Teflon mold and left to gel at room temperature for 10 min. Next, a CHG specimen (4 mm diameter × 2 mm thickness) was immediately placed on top of the freshly formed fibrin gel, followed by the addition of another 250 μL of the pre-mixed fibrinogen/thrombin/CaCl_2_ solution to cover the CHG specimen. The entire material was incubated at 37 °C for 30 min. Afterward, the CHG-embedded fibrin gel (encoded as FCHG) attached firmly to the Eppendorf tube was removed from the Teflon mold and placed into a 24-well plate for subsequent experiments. The whole preparation process was schematically illustrated in Scheme 1b.

To investigate whether GGOH had an interaction with fibrinogen upon the preparation of CHG-embedded fibrin gel, which could affect the prime release efficiency of GGOH from FCHG, fluorescence spectroscopy was in use for observing any changes in spectral intensity and/or peak wavelength in a mixed GGOH/fibrinogen/thrombin/CaCl_2_ solution, compared to that of a mixed fibrinogen/thrombin/CaCl_2_ solution. Briefly, 1 mL of 1 mg/mL fibrinogen mixed with thrombin and CaCl_2_ in Milli-Q water was placed in a quartz cuvette in the presence or absence of 50 μg of GGOH dissolved in 20 μL of EtOH. The fluorescence emission spectrum (290–500 nm) of each solution was immediately collected at room temperature by means of a spectrofluorometer (Model FP 8500, Jasco International) using excitation (λ_ex_) at 280 nm with 1 nm-width excitation.^15^ 1 mL of a mixture of 980 μL of Milli-Q water and 20 μL of GGOH/EtOH solution (containing 50 μg of GGOH) was also concurrently analyzed.

### 2.5 Characterization of Morphologies and Structures of Porous Hydrogels

The surface morphologies of pure fibrin gel (F) and dual drug-loaded hydrogel (CHG) specimens coated with gold in a sputtering device were examined by a scanning electron microscope (SEM) (Hitachi S-3400N, Japan, 15 kV of an accelerating voltage). The average pore sizes of F and CHG were measured directly from their SEM images by ImageJ (U. S. National Institutes of Health, Bethesda, Maryland, USA) using 50 pores per image (*n* = 2). Pore structure and microstructural morphology of the dual drug-loaded hydrogel embedded in fibrin gel (FCHG) were analyzed in comparison with those of CHG using X-ray microcomputer tomography (μCT). The freeze-dried specimens were scanned using a μCT SkyScan 1275 (Bruker μCT, Kontich, Belgium) under the following parameters: pixel size = 8 μm, source voltage = 40 kV, source current = 80 μA, no filter and rotation step = 0.2°. Visualizations were acquired using a DataViewer (2D cross-section images, Bruker) and a CTVox (3D images, Bruker). The datasets were binarized using an adaptive threshold to distinguish dense material regions from voids, and despeckle operations in 3D were applied to reduce image noise. Analysis of porosity was performed by means of a CTAn (Bruker), and the interconnectivity was calculated as a percentage of the volume of open pore space to the total volume of pore space. The total porosity (%), interconnectivity (%), closed porosity (%), and the number of closed pore data are obtained as mean ± SD based on the analysis of middle and peripheral regions (thickness = 1 mm) of a hydrogel specimen.

To observe the penetration of fibrin into CHG, autofluorescence of the fibrin matrix was employed. The CHG specimens were first soaked in 0.4% trypan blue solution for 10 min at room temperature and washed three times with phosphate buffer saline (PBS) to eliminate the autofluorescence signal from CHG. They were then individually soaked in water, human plasma, or human plasma gel (CaCl_2_ added at a final concentration of 10 mM) for 30 min at 37 °C before imaging. The plasma (gel) at the bottom of each plasma (gel)-coated specimen was wiped before scanning using a Nikon C2plus confocal microscopy. The specimens were captured using S Plan Fluor ELWD 40× objective in two channels (green and blue). A selection of micrographs of the specimens was captured, consisting of 35 z-slices with a step size of 1.9 μm. The gain settings were kept the same for all data acquisition. Green channel fluorescence imaging at 488 nm excitation and 525/50 nm detection, as well as blue channel fluorescence imaging at 405 nm excitation and 447/60 nm detection, were used to obtain the merged dual color autofluorescence images.

### 2.6 In Vitro Drug Release Behaviors of CHG and FCHG

In brief, a CG-loaded composite hydrogel (CHG) specimen (3.5 ± 0.1 mg) or a CHG-embedded fibrin gel (FCHG) specimen clung to the Eppendorf tube had been immersed in 1.2 mL of either a PBS-based mixed fluid (coded as PBB), containing PBS mixed with 10% fetal bovine serum (FBS), or a simulated interstitial fluid (coded as SIF), containing simulated body fluid (SBF) mixed with 10% human serum, at 37 °C for 14 days. The SIF preparation procedure is described in the Supporting Information (SI) and Table S1. A 1 mL aliquot of the supernatant was collected daily for the measurements of amounts of CDM and GGOH released from the specimen by high-performance liquid chromatography (HPLC; WATERS HPLC 2965 SYSTEM). After liquid collection, 1 mL of fresh releasing medium was immediately added to keep the volume of the test specimen constant. To prevent HPLC column clogging, deproteinization of each collected supernatant was performed prior to HPLC sample injection using the following protocols.

#### 2.6.1 HPLC Samples for CDM Analysis

The double liquid extraction process was used by following the process previously reported by Batzias et al.^16^ Typically, 0.5 mL of each collected supernatant was mixed with 1 mL of acetonitrile with the use of a vortex mixer for 1 min and then centrifuged at 10,000 rpm for 5 min. The supernatant was then transferred into a new tube containing 50 μL of 0.4 M sodium hydroxide and mixed for 5 min to allow the formation of a non-ionized extractable form. Then, 6 mL of dichloromethane was added, followed by vortex mixing for 5 min and centrifuging at 10,000 rpm for 10 min. The lower aqueous layer was collected and evaporated at ambient temperature until all solvents were depleted. The remaining residue was subsequently redissolved in the HPLC mobile phase for CDM analysis.

#### 2.6.2 HPLC Samples for GGOH Analysis

Typically, 0.5 mL of each collected supernatant was vortex mixed with 1 mL of cold ethanol to precipitate out the protein and then centrifuged at 10,000 rpm for 10 min. The clear supernatant was collected for HPLC analysis.

All the collected deproteinized samples were individually filtered using 0.45 μm PTFE membranes prior to HPLC injections (HPLC experimental conditions used are displayed in Table S2). The amount of CDM or GGOH found in each collected specimen was quantified against the standard curve of each drug which was constructed from a series of serial dilutions of known CDM or GGOH solution concentrations which were prepared in the corresponding releasing medium and subsequently deproteinized using the relating protocol stated above. The cumulative release of the drug was calculated by the following equations (eqs 1 and 2).
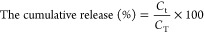
1
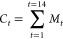
2where *C_t_*, *C*_T_, and *M_t_* represent the cumulative drug released at day *t* (*t* = 1–14), the total amount of drug loaded in the material, and the amount of drug released at day *t* (*t* = 1–14) (*n* = 2).

### 2.7 In Vitro Drug Release Kinetics of CHG and FCHG

To study the release mechanisms of CDM and GGOH from the CG-loaded composite hydrogels (CHG) and CHG-embedded fibrin gel (FCHG), the release data of both drugs were individually fitted to the following mathematical models (eqs 3–7). The linear regression plots using OriginPro software (OriginLab Corporation, MA, USA) were applied to all the drug release profiles.
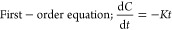
3where *C* is the concentration of drug released at the time (day) *t*; *K* is a first-order rate constant, and *t* is the release time (day).^17^
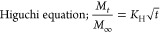
4where *M*_t_ and *M*_∞_ are the cumulative drug releases at day *t* and infinite day, respectively; *K*_H_ is a Higuchi dissolution constant, and *t* is the release time (day).^18^
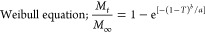
5where *M*_t_ and *M*_∞_ are the cumulative drug releases at day *t* and infinite day, respectively; *a* and *b* are the time scale of the process and the shape parameter, respectively; *T* is the lag time before the onset of the release process (in most cases will be zero), and *t* is the release time (day).^19^
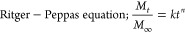
6where *M_t_* and *M*_∞_ are the cumulative drug releases at day *t* and infinite day, respectively; *k* is a release kinetic constant; *t* is the release time (day), and *n* is the release exponent which indicates the drug release mechanism.^20^
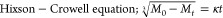
7where *M*_0_ represents the initial amount of CDM or GGOH loaded in the hydrogel; *M_t_* is the cumulative drug releases at day *t*; κ is a constant incorporating the surface volume relation, and *t* is the release time (day).^21^

### 2.8 In Vitro Antibacterial Activity of CHG and FCHG

The antibacterial activity of CHG and FCHG was determined against *Streptococcus sanguinis* (ATCC 10556, ATCC, Manassas, USA) using the protocol as previously described.^8^ Briefly, the bacteria were cultured in a brain heart infusion (BHI) broth, and a 1.2 mL bacterial suspension (3 × 10^5^–7 × 10^5^ CFU/mL) was exposed to a test hydrogel for 24 h. The total number of viable and active bacteria that remained in each sample was ultimately measured in CFU. The antibacterial activity of the test hydrogel on a given incubation day was calculated using the following equation.
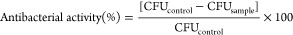
8where CFU_control_ and CFU_sample_ are the colony-forming units found in the bacterial solutions without and with the test hydrogel, respectively (*n* = 2).

In some experiments where the growth of bacteria was not clearly observed in the suspension, the number of bacteria found after 24 h incubation with the test specimen was estimated by measuring the optical density (OD) at a wavelength of 600 nm (OD600) using a spectrophotometer. The bacterial growth (%) was calculated from the OD600 value with respect to that of the bacterial suspension without any test hydrogel which was defined as 100%. To confirm the bactericidal activity of the test specimen, the drop plate method was used to examine the presence of viable suspended bacteria.

### 2.9 In Vitro Cytoprotective Activity of CHG and FCHG against ZA

Human mesenchymal stem cells (MSCs; Lonza Biologics plc, Cambridge, UK) at passages 5–8 were maintained in a standard medium: α-minimum essential medium (α-MEM) (Gibco Life Technologies Ltd., Paisley, UK) containing 10% FBS supplemented with 200 U/mL penicillin, 200 μg/mL streptomycin, and 2 mM l-glutamine (all from Gibco) at 37 °C under 5% CO_2_ atmosphere. The UV-sterilized CHG specimens were pre-incubated in the culture medium for 24 h before all cytoprotection experiments, while the FCHG120 specimens were those taken from the 14 day incubation of FCHG120 in SIF and subsequently further incubated in cell culture for 7 days.

For the cytoprotection test, MSCs at a density of 3 × 10^4^ cells/cm^2^ were plated in 24-well plates and cultured for 18 h before exposure to 5 μM ZA (Aclasta, Novartis Pharmaceuticals UK Ltd., UK), with each hydrogel specimen being placed in the upper compartment of a semi-permeable porous membrane (0.4 μm) cell culture insert (Nunc, VWR Ltd., Lutterworth, UK). A schematic diagram of the culture with the drug-loaded hydrogel is shown in Figure S1. For all experiments, the volume of culture medium per well was 1.2 mL, with the medium being refreshed every 2 days. The cells were cultured for indicated times before cell morphology and viability analysis.

The protected MSCs that survived ZA cytotoxicity were further assessed to determine whether their osteogenic differentiation potential remained preserved. Surviving MSCs were re-plated at a density of 1.5 × 10^4^ cells/cm^2^ in 24-well plates and allowed to grow in the standard culture medium for 48 h. Then, the cells were incubated with an osteogenic medium (OM) (standard medium with 100 nM dexamethasone, 50 μM ascorbate-phosphate, and 10 mM β-glycerolphosphate, all from Sigma) for 7 days (for gene expression assay) and 21 days (for mineralization assay).

#### 2.9.1 Cell Morphological Analysis and Assessment of ZA Cytotoxicity Reversal Ability

The cells were fixed with 4% paraformaldehyde (PFA) and stained with 0.05% (w/v) crystal violet solution after the stated durations in the medium of the specimens. A light microscope was used to evaluate the cell morphology (Nikon Eclipse TS100, Nikon Instruments Inc., NY, USA). Meanwhile, the cell samples were subjected to the 3-(4,5-dimethylthiazol-2-yl)-2,5-diphenyltetrazolium bromide (MTT) test. The end product was then tested for absorbance at 490 nm (A490), which is proportional to the viability of cells. The reversal of ZA cytotoxicity was calculated using the equation below.
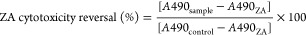
9where *A*490_sample_, *A*490_control,_ and *A*490_ZA_ are the absorbances measured from MSCs treated with ZA and a hydrogel, untreated MSCs, and MSCs treated with ZA only, respectively.

#### 2.9.2 Quantitative Real-Time Reverse Transcription-Polymerase Chain Reaction (qPCR)

Total RNA was extracted from each sample using the RNeasy Mini Kit (Qiagen, West Sussex, UK) according to the manufacturer’s instructions. For the reverse transcription reaction, 1 μg of total RNA was used to synthesize the first strand of cDNA, after which 1 μL of each cDNA sample was subjected to qPCR using SYBR Green I dye. The specific primers for runt-related transcription factor 2 (RUNX2), alkaline phosphatase (ALP), type-I collagen (COL-I), and glyceraldehyde 3-phosphate dehydrogenase (GAPDH) mRNA were used in the PCR reactions (Table S3).^22,23^ All PCR reactions were performed in six replicates, and each of the signals was normalized to the GAPDH signal in the same reaction.

#### 2.9.3 Mineralization Assay

Mineralization was measured by an alizarin red S assay. After 21 days in osteogenic induction culture, the cultured cells were fixed with ice-cold methanol and stained with 1% alizarin red S (pH 4.2; Sigma). Incorporated alizarin red S was extracted by the addition of 100 mM cetylpyridinium chloride (Sigma), and the absorbance (A570), which is proportional to the alizarin red S-positive mineralization, was measured.

### 2.10 In Vitro Acute Cytotoxicity of CHG and FCHG

Murine monocyte/macrophage RAW 264.7 cells (RAW cells; ATCC) were used for the cytotoxicity test as they represent acute inflammatory cells. RAW cells were maintained in a standard medium at 37 °C under a 5% CO_2_ atmosphere. A 1.2 mL cell suspension (1 × 10^6^ cells/mL) was exposed to the hydrogel test specimen in a 15 mL polypropylene conical tube (Corning, NY, USA) for 48 h. The samples were then stained with propidium iodide (PI; 1 μg/mL) and analyzed by flow cytometry. The level of PI-positive cells corresponds to cytotoxicity.

### 2.11 In Ovo Chick Embryo Chorioallantoic Membrane (CAM) Model

The model was used to determine the effect of the fibrin matrix on the possible adverse effect on angiogenesis and the acute toxicity of the CG-loaded composite hydrogel. Fertilized chicken eggs (embryo development day 1, ED1) of the ISA Brown chicken were kindly provided from CPF (Thailand) PCL., Thailand. The eggs were disinfected with ethanol and then incubated in a temperature/humidity-controlled incubator at 37 °C and 65% relative humidity. On ED3, a square window sized 1 × 1 cm^2^ on the eggshell was made and then sealed with adhesive tape to avoid contamination and desiccation of the egg contents. The eggs were then further incubated in horizontal setters. A specimen was rehydrated with PBS and gently placed into an ED5 egg (for angiogenesis) and an ED7 egg (for acute toxicity). After 48 h incubation, the vascularized CAMs of eggs (ED7) were photographed while the chick embryos (ED9) were fixed with 10% formalin for 24 h and photographed. The experiments were performed in four replicate eggs/group/assay. Vascular density (expressed as %area) and vascular branching (number of vascular branches/area) were analyzed using the Vessel Analysis plugin with Fiji (ImageJ) software version 1.47 g. The death of embryos, evident by their motionless, and the growth retard of the viable embryo at 48 h post-implantation of the samples were considered acute toxicity.^24^

### 2.12 Statistical Analysis

The studies were carried out in at least triplicate unless otherwise noted, and the results were given as the mean ± SD based on three separate experiments. Using a one-way ANOVA and post hoc Bonferrini’s test, statistical differences were examined using SPSS software (SPSS, Inc., Chicago, IL). A *p*-value of 0.05 or less was considered statistically significant.

## 3 Results and Discussion

Upon implantation, a drug-loaded carrier unavoidably contacts with blood. When a blood clot forms and surrounds the implanted material, the release behaviors and kinetics of drugs loaded in the carrier can be altered. In the present study, not only the as-produced CG-loaded composite hydrogels, i.e., CHG60 and CHG120 prepared, but also the human-derived fibrin-surrounded CHG120 hydrogel, i.e., FCHG120, was prepared to generate a more physiological-like in vitro model mimicking tissue matrices. The drug release profiles and the physical and biological properties of these two different CG-loaded hydrogels were comparatively evaluated to elucidate how the performance of the CHG hydrogel would be affected if it were to be enclosed in a fibrous clot, particularly for the prevention of MRONJ associated with ZA.

### 3.1 Fabrication and Characterization of Porous Hydrogels

**Figure 1:**
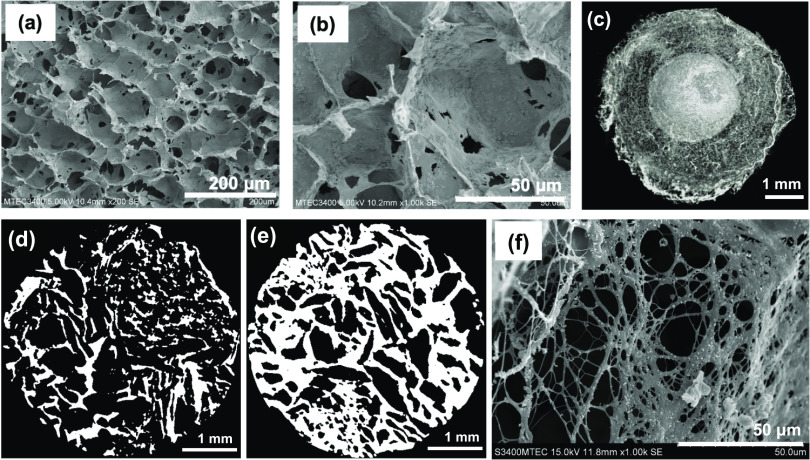
Pore structures and microstructural morphologies of prepared porous hydrogels. SEM images of surface morphology of CHG120 at 200× (a) and 1000× (b). 2D reconstructed μCT images of FCHG120 (c), as-produced CHG120 (d), and CHG120 embedded in fibrin network (e). SEM image of surface morphology of fibrin network at 1000× (f).

The pore structure, e.g., size, porosity, and interconnectivity, of a porous drug-loaded carrier plays an important role not only in the drug release profile but also in the new tissue in-growth.^25^ As reported in our previous study,^8^ the impregnation of 420 mg of PMCM-41 in the solution of 200 mg of CDM in 3 mL of DI water at ambient temperature for 24 h yielded the nanoparticles loaded with approximately 30% by weight of CDM (coded as PMC), determined by TGA-DTA. PMC was found to prolong the release of CDM in PBS for at least 10 days. The lyophilized CDM-loaded sponge-like composite pad (10 × 7 × 0.2 cm^3^) was subsequently prepared from the mixture of 630 mg of CM powder dissolved in 7 mL of DI water and 420 mg of PMC dispersed in 3 mL of DI water (weight ratio of CM to PMC = 60:40) and then subjected to the steam-induced crosslinking at 105 °C for 10 min before being cut into disks (4 mm diameter × 2 mm thickness). In this present study, 60 or 120 μg of GGOH in EtOH was subsequently loaded dropwise onto the CDM-loaded hydrogel disks to generate a CG-loaded composite hydrogel (CHG), namely CHG60 or CHG120, respectively, as illustrated in Scheme 1a. The CHG120 hydrogel was later brought in contact with the pre-mixed fibrinogen/thrombin/CaCl_2_ solution to form the CHG-embedded fibrin gel (FCHG) by using the preparation process schematically, as shown in Scheme 1b. The surface morphology of CHG120 examined by SEM exhibited an interconnected porous structure of the hydrogel with the PMC agglomerates intermittently distributed in its matrix, as shown in Figure 1a,b. The average pore size of the hydrogel measured directly from the SEM image using 50 pores per image (*n* = 2) by ImageJ was about 90 ± 34 μm. The pore structure of CHG120 fairly resembled that of the CG-free composite hydrogel, which was previously reported in the literature,^8^ suggesting that preloading of CDM into PMCM-41 prior to the fabrication of the composite hydrogel and post-loading of GGOH onto the fabricated CDM-loaded composite hydrogel scarcely changed the morphological structure of the porous composite hydrogel.

After the encapsulation of CHG120 in the human-derived fibrin gel, the microstructural morphology and pore structure of the resulting hydrogel, i.e., FCHG120, were examined in comparison with those of the starting CHG120 specimen using μCT. The 2D reconstructed μCT image of FCHG120 explicitly showed the microstructure of CHG120 surrounded with a fine mesh of long thin fibrin fibers (Figure 1c). The total porosity values of both starting CHG120 and CHG120 embedded in the fibrin gel were comparatively determined from their 3D reconstructed μCT images. Noteworthily, the % porosity of FCHG120 became subsided from 75 ± 0.7% (% porosity of starting CHG120) to 36 ± 22.6%, indicating that, upon the fibrin formation, CHG120 was soaked with the fibrinogen/thrombin/CaCl_2_ solution and then partially filled with the entangled network of fibrin fibers, which was observed more densely around the peripheral region of CHG120, as seen in the binary μCT images in Figure 1d,e. In FCHG120, the decrease in the porosity of CHG120 embedded was also accompanied by a minimal increase in the number of closed pores of the dual drug-loaded hydrogel. Nevertheless, the interconnectivity of the hydrogel partially filled with fibrin was barely perturbed (98.5% vs 99.9% for the starting CHG120). This would reasonably allow the sufficient diffusion of oxygen/nutrients and the capillary in-growth to the implanted CHG120 at the tooth extraction site in vivo. Meanwhile, the human-derived fibrin gel was also formed using the same procedure, as illustrated in Scheme 1b with no added CHG120. The high-magnification SEM image (×1000) shown in Figure 1f reveals a fine mesh-like loose structure of fibrin fibers with pore sizes in the range of 2–30 μm, analyzed by ImageJ using 50 pores per image (*n* = 2).

**Figure 2:**
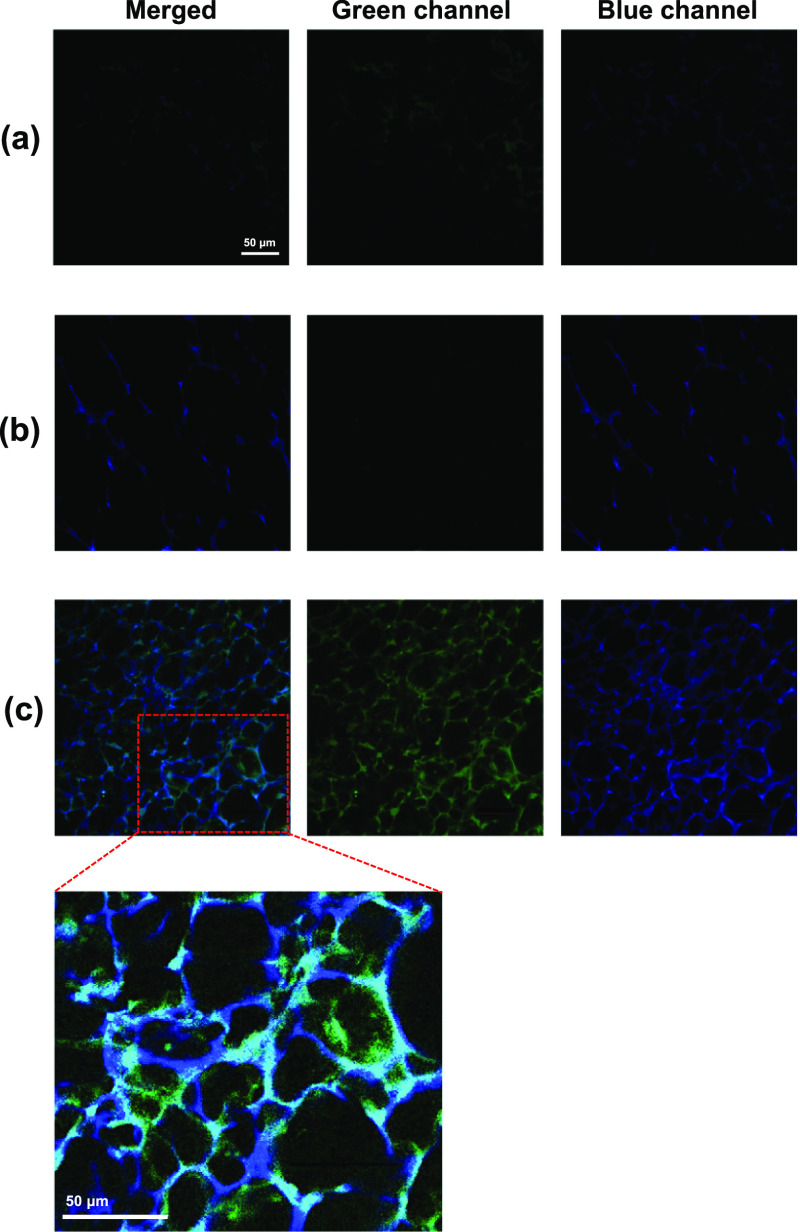
Confocal microscopy images (40× objective) of autofluorescence of CHG120 after being soaked in water (a), human plasma (b), or human plasma gel (c), where green (fibrin-containing matrix) and blue (plasma proteins) emission channels were captured at 19 μm depth with the same microscopy setting. The close-up image of the merged image in (c) revealed the presence of a fibrin-containing network (green) in the porous pores and along the porous walls of the hydrogel, together with plasma proteins (blue) being adsorbed on the hydrogel.

As reported in the literature, fibrin fibers were shown to possess green autofluorescence properties,^26,27^ while several components in plasma such as protein-bound tryptophan and enzyme-bound NAD(*P*)H exhibited strong autofluorescence in the blue emission channel.^28^ Fibrin formed in the pores of CHG120 was further observed by confocal microscopy imaging analysis of autofluorescence of CHG120 after being soaked in water (used as a control), human plasma, or human plasma gel (CaCl_2_ added) for 30 min at room temperature. The porous structure of the CHG120 control group showed low signals of autofluorescence in both green and blue channels (Figure 2a), while the autofluorescence of the plasma-soaked CHG120 was displayed only in the blue channel, plausibly derived from some plasma protein aggregates within the CHG120 pores (Figure 2b). Bright autofluorescence in both green and blue channels was notably detected in the pore structure of the plasma gel-soaked CHG, indicating the co-existence of a fibrin network and plasma protein aggregates in the hydrogel pores (Figure 2c). This observation was wholly in agreement with the decrease in the porosity of the dual drug-loaded composite hydrogel after being encapsulated in the human-derived fibrin gel, analyzed by μCT.

**Figure 3:**
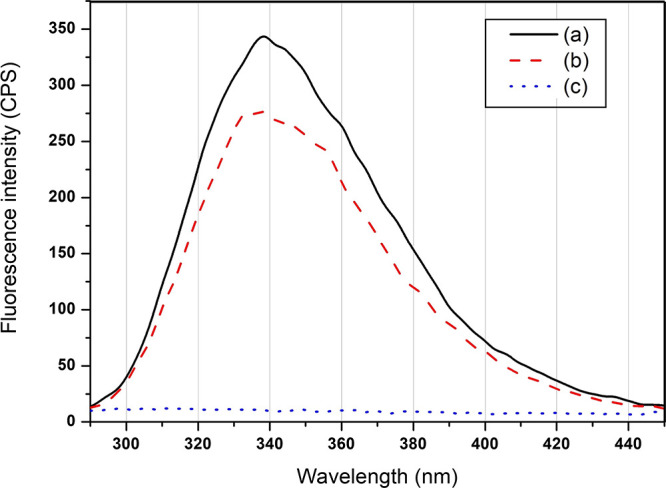
Fluorescence spectra (λ_ex_ = 280 nm) of fibrinogen in the fibrinogen/thrombin/CaCl_2_ solutions mixed without (a) and with (b) 50 μg of GGOH dissolved in 20 μL of EtOH and GGOH in the mixture of Milli-Q water/GGOH/EtOH solution (c).

It was of interest to investigate whether GGOH, a poorly water-soluble monoterpenoid alcohol, could interact with proteins, especially fibrinogen, during blood clotting. Should any intermolecular interactions occur, the release behavior of GGOH from FCHG120 will be consequently different from that of the starting CHG120. To prove this, the fibrinogen/thrombin/CaCl_2_ solution mixed with and without 50 μg of GGOH dissolved in 20 μL of EtOH and the GGOH/EtOH solution were employed as models in the investigation of the interaction between GGOH and fibrinogen upon the preparation of FCHG120. The fluorescence emission spectra of all three solutions were immediately gathered at room temperature and then overlaid, as illustrated in Figure 3. The spectrum of fibrinogen collected from the fibrinogen/thrombin/CaCl_2_ solution possessed the maximum λ_em_ at ∼338 and 345 nm with a broad spectrum extending from 290 to 450 nm (Figure 3a). It is noteworthy that the fluorescence intensity of fibrinogen was slightly lessened when GGOH was added to the fibrinogen/thrombin/CaCl_2_ solution, as depicted in Figure 3b. This fluorescent quenching clearly suggested the partial binding between fibrinogen and GGOH molecules, leading to non-fluorescence complex formation, because no fluorescence emission spectrum was detected from the GGOH/EtOH solution (Figure 3c). This observation agreed with a previous study by Gonçalves et al.,^15^ who reported that the interaction of β-estradiol, having non-ionized and lipophilic molecules, with fibrinogen macromolecules could induce the conformational changes in the protein structures and cause the quenching of fluorescence.

### 3.2 In Vitro Release Behaviors of CDM and GGOH from Porous Hydrogels

**Figure 4:**
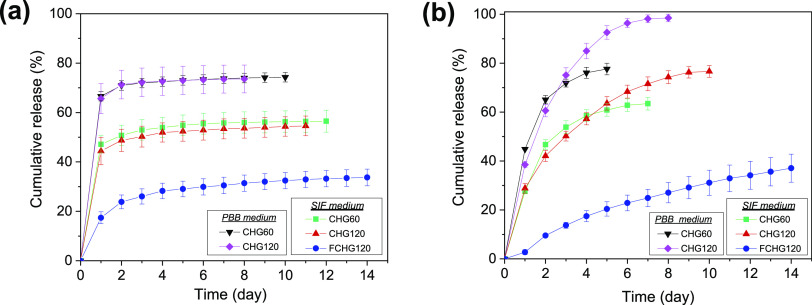
Cumulative amount of drug released versus time: CDM (a) and GGOH (b). The amount of drug released daily was quantified by HPLC (*n* = 3) after individual CG-loaded composite hydrogels, surrounded with or without a fibrin network, were incubated in either PBB or SIF at 37 °C for 14 days.

The in vitro release behaviors of each drug loaded in CHG60 and CHG120 were comparatively studied in two different releasing media, i.e., PBB and SIF. SIF was exploited to imitate the interstitial fluid, a bodily fluid essentially produced via trans-capillary blood exchange where cells and tissues are surrounded.^29^Figure 4a presents the cumulative releases (%) of CDM from the hydrogel disks (3.5 ± 0.1 mg), loaded with a fixed amount of CDM (about 30% by weight of PMCM-41 which was integrated into the hydrogel at 40% by weight) and two different contents of GGOH (60 and 120 μg/hydrogel, presumably no losses of GGOH upon loading), in PBB and SIF as a function of immersion time. Notably, the initial burst releases of CDM from CHG60 (66%) and CHG120 (67%) were observed on Day 1 when they were separately incubated in PBB, followed by slow plateau releases of CDM lasting up to 10 days with the total CDM releases of 74 and 73%, respectively. The considerable initial releases of CDM were likely attributed to the fast diffusion of the hydrophilic drug, particularly that localized in the interconnected porous hydrophilic CM matrix of hydrogel, which was resulted from the leaching of CDM weakly adsorbed at the surface of PMCM-41 into the polymer solution, upon the preparation of CDM-loaded composite hydrogel. Apparently, the quantity of GGOH loaded into the hydrogels determined the release behavior of CDM, specifically the sustained-release period. CHG60 could sustain the release of CDM two days longer than CHG120, up to 10 days. The co-existence of GGOH in the hydrogels plausibly somewhat impeded the release of CDM, not only was the release time shortened in CHG120 but also the sum amount of CDM released from this hydrogel was slightly lessened, as shown in Table S4. As reported earlier, the amount of CDM initially liberated from the CDM-loaded composite hydrogel incubated in PBS at 37 °C on Day 1 was about 243.5 ± 11.6 μg/mL with the cumulative amount of CDM freed in 10 days of 334.4 ± 14.9 μg,^8^ which were relatively greater than those of CHG60 and CHG120, totally confirming that the less hydrophilic GGOH layers located exclusively in the hydrophilic polymer matrix of hydrogel slightly hindered the diffusion of CDM into the releasing medium. At last, after 14-day incubation of the hydrogels in PBB, there seemed to be about 26–27% of CDM still tightly bound in the hydrogels, particularly in the porous nanoparticles sporadically distributed throughout the porous polymer matrix, as well as in the matrix itself, as it was noted that CM could form a chemical linkage with CDM upon the steam crosslinking of the freeze-dried hydrogel pad,^8^ unless there were releases of the drug after Day 8 (for CHG120) and Day 10 (for CHG60) at concentrations below the detection limit of HPLC used in this study.

The release patterns of CDM from both CHG60 and CHG120 immersed in SIF quite resembled those perceived from the specimens submersed in PBB, except that the burst CDM releases were drastically lessened from 66–67% (in PBB) to 44–47% (in SIF) with slightly longer release periods from 8–10 days (in PBB) to 11–12 days (in SIF). Moreover, the cumulative amounts of drug released found in the aliquots collected from the 14 day incubation of CHG specimens were considerably lowered from 73–74% (in PBB) to 56–57% (in SIF). These all were associated with the hydrophobicity of human serum-containing SIF, which was higher than that of bovine serum-containing PBB,^30^ further declining the release rates of CDM from the composite hydrogels, aside from the partially hindered diffusion of CDM through the less hydrophilic GGOH layers in the hydrogel matrix before entering into the medium. Since the release of CDM was evidently obstructed by both the presence of a co-loaded less hydrophilic drug (GGOH) and the usage of a much less hydrophilic releasing medium (SIF), a great deal of CDM (almost 50%) was still inevitably confined in the hydrogels after being incubated for 14 days. When the CG-loaded composite hydrogel is to be implanted in the body, the more controlled release of CDM from the material will be enabled in the surrounding interstitial fluid. All tissues nearby the implanted material will be more prolongedly treated with an initial exposure to a less excessive dose of the antibiotic.

Figure 4b demonstrates the cumulative releases (%) of GGOH from CHG60 and CHG120 incubated in PBB and SIF as a function of time. Overall, the release profiles of GGOH of these two hydrogels in PBB were rather alike with the amounts of GGOH initially released on Day 1 about 39 and 45%, sustained-release periods of 5 and 8 days, and total amounts of drug release of 78 and 98%, respectively. Fascinatingly, GGOH was progressively liberated from the composite hydrogels for at least 5 days with much lower initial burst releases observed on Day 1, compared to those of CDM, indicating that the diffusion of GGOH through the hydrophilic CM matrix was more difficult than that of CDM. Apparently, the difference in the hydrophilicity of a drug loaded and the matrix of a carrier could readily enable the controlled release of the drug. GGOH loaded in the hydrogels was, however, nearly completely freed from CHG60 and CHG120 (about 78 and 98% within 5 and 8 days, respectively), implying that both CM and PM were scarcely able to retain GGOH.

Like those of CDM, not only was the release rate of GGOH reduced but also the sustained-release period of GGOH was slightly prolonged when the hydrogels were individually incubated in SIF. The initial GGOH releases on Day 1 from CHG60 and CHG120 were found to be only 28 and 29%, respectively, with the total amounts of GGOH liberated of 63% (in 7 days) and 77% (in 10 days), respectively, suggesting that the hydrophobicity of SIF could be higher than that of GGOH. Interestingly, SIF, an interstitial fluid-like releasing medium, not only declined the release rates of CDM and GGOH but also slightly prolonged the sustained-release periods of both drugs. This finding was beneficial to the further study of these CG-loaded composite hydrogels when their release profiles will be evaluated in vivo.

To further investigate the release behaviors of the drugs loaded in the composite hydrogel in a more physiological-like in vitro model, CHG120 was brought into contact with the fibrinogen/thrombin/CaCl_2_ solution, generating a CHG120-embedded human-derived fibrin gel (FCHG120), which more or less mimicked the situation when a drug-loaded carrier was surrounded with a blood clot after implantation. The release profiles of CDM and GGOH loaded in FCHG120 were studied in comparison with those of CHG120 in SIF to understand how the closely imitated in vivo environment would affect the drug deliverability of the hydrogel. It was noteworthy that the initial burst release of CDM observed on Day 1 in FCHG120 was reduced by more than half of that found in CHG120, only 17% of CDM was liberated from the hydrogel when it was enclosed in the fibrin network (Figure 4a). More interestingly, CDM was more progressively and lengthily freed from FCHG120, compared to that in CHG120. CDM had been gradually released for at least 14 days with the sum of drugs released of only 34% (Table S4 and Figure 4a). This significantly altered release profile was explicitly associated with the presence of porous fibrin fibers, which were located not only outside the hydrogel but inside the porous structure of CHG120, evidenced by μCT analysis (Figure 1e) and confocal microscopy (Figure 2). The double-matrix structure of FCHG120, as well as the physical entanglement of fibrin and hydrogel matrix within the hydrogel pores absolutely hindered the liberation of CDM from the nanoparticles and the diffusion of CDM from the polymer matrix of the hydrogel. It was previously reported that the in vitro release of recombinant human epidermal growth factor (rhEGF) encapsulated in chitosan (CS) nanoparticles became more sustained when the rhEGF/CS nanoparticles were incorporated in fibrin gels that were prepared from varied contents of fibrinogen and thrombin via a dual-chamber syringe injection of fibrinogen reconstituted in the potassium dihydrogen phosphate solution, added in a syringe, and thrombin reconstituted in CaCl2 solution mixed with rhEGF/CS nanoparticles, loaded in another syringe.^31^ Rather similar to that of CDM, the release profile of GGOH from FCHG120 was considerably shifted from that observed from CHG120. The quantity of GGOH liberated from FCHG120 in SIF on Day 1 was astonishingly only 3% (Figure 4b). In addition, its prolonged release, up to at least 14 days, appeared nearly linear-like, with the total content of GGOH released of only 37%. Undoubtedly, the presence of fibrin gel in FCHG120 markedly slowed down the diffusion of GGOH mainly localized in the CM matrix into the releasing medium. However, the almost linear-like drug release profile was not solely caused by the dual matrices of FCHG120 and the physical entanglement of the fibrin and hydrogel matrix. A substantial amount of GGOH (about 63%) was still not unleashed. As observed by fluorescence spectroscopy, there existed a partial intramolecular interaction between GGOH and fibrinogen molecules (Figure 3). During the preparation of FCHG120, GGOH molecules that diffused from the CM matrix and got into the fibrinogen/thrombin/CaCl_2_ solution could partly form an intermolecular interaction with fibrinogen molecules and subsequently be parts of the synthesized fibrin gel, making them difficult to be leached out of the fibrin network. This could predominantly suppress the release rate of GGOH formerly found in CHG120. Taken all together, under the whole circumstances, the deterioration in the release rates of drugs loaded in a carrier must be cautiously considered whenever the material will be brought into contact with substances that have entirely different physiochemical properties and structures.

### 3.3 In Vitro Release Kinetics of CDM and GGOH from Porous Hydrogels

A successful drug delivery system can be essentially achieved when a drug carrier and a drug-loading process are both optimally designed to enable a controlled and/or sustained drug release. The release mechanism is normally studied through experimental verification using mathematical modeling. There are, in general, several factors that determine the drug release kinetics, such as type and composition of a carrier, type, and composition of a releasing medium, and the interaction between a carrier and a loaded drug.^32^ In this study, five mathematical models, i.e., first-order, Higuchi, Weibull, Ritger–Peppas, and Hixson–Crowell models (see their equations in Section 2.7), for dual drug delivery system, were individually assessed to describe the kinetics of CDM and GGOH releases from the porous hydrogels.

**Figure 5:**
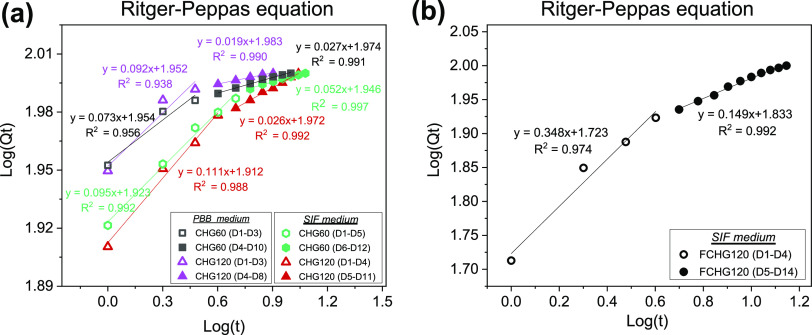
Release kinetics of CDM from as-produced CHG hydrogels (a) and FCHG120 (b) after being incubated in PBB and SIF for 14 days. The linear regression plots were constructed from the acquired drug-releasing profiles and fitted by the Ritger–Peppas equation.

Among all mathematical models used, the Ritger–Peppas equation best fitted the whole-lifetime release profiles of CDM from the CHG hydrogels surrounding with and without fibrin network. The drawn linear regression plots with the highest values of the regression coefficient (*R*^2^) ranging from 0.938 to 0.997 are shown in Figure 5 and Tables S6 and S7. According to Ritger–Peppas model, the kinetics of drug releases with diffusion mechanisms was essentially described by Fick’s law. The release exponent (*n*) and the slope of a plot, demonstrates the release mechanism of a drug from a cylindrical-shaped (disc) carrier in four different phenomena, i.e., *n* < 0.45 for quasi-Fickian diffusion, *n* = 0.45 for Fickian diffusion, 0.45 < *n* < 0.89 for non-Fickian diffusion or anomalous transport, and *n* = 0.89 for Case-II transport.^33^ Interestingly, the whole-lifetime release patterns of CDM appeared nondifferentiable, divided into two releasing stages, i.e., rapid (burst) and steady releasing stages. CDM released in the first stage was primarily the drug that initially leashed out of PMC and then diffused into the CM matrix during the preparation of CDM-loaded composite hydrogel pads, while that steadily liberated in the second stage was the drug that was gradually freed from porous PMC through the hydrogel matrix before entering the surrounding medium. The nondifferentiable release pattern, fitted by the Ritger–Peppas equation, was plausibly associated with the small *n* values, found in the range of 0.019–0.348, suggesting that the releases of CDM from the hydrogels occurred through a quasi-Fickian diffusion process with physico-chemical interferences. There existed some physical interferences between CDM and such surroundings as the relatively less hydrophilic GGOH layers located mainly in the hydrogel matrix and the relatively more hydrophobic releasing media (both PBB and SIF), both of which could hinder the transport and diffusion of water-soluble CDM.

The determined *n* values of CDM released from both CHG60 and CHG120 soaked in PBB and SIF at each releasing stage seemed insignificantly different, as revealed in Figure 5a. The release of CDM essentially involved the diffusion from the hydrogel into the external environment. However, the durations of the rapid and slow drug-releasing stages were rather different when the materials were surrounded by different fluids. In SIF, containing more hydrophobic proteins, as well as ions, which yielded a poor driving force for CDM release, both releasing stages became extended, compared to those of the hydrogels incubated in PBB. The burst releasing stage of CDM in SIF was extended for 1–2 more days, while the slow drug-releasing stage caused by the drug concentration gradient was prolonged for 2–3 more days. Though the release kinetics of CDM from CHG120 and FCHG120 appeared rather similar, perfectly fitted by the same mathematical model, the *n* values observed in FCHG120 at both releasing stages were much greater than those obtained in CHG120. Such a high *n* value (0.348) in the first releasing stage, as shown in Figure 5b, suggested that most of the CDM initially released from FCHG120 by the diffusion process was the drug located in the fibrin gel. During FCHG120 formation, CDM freely localized in the CM matrix of CHG120 leached out and then diffused into the fibrin network where existed no physical interference between CDM and fibrin. The surrounding fibrin network, however, further sustained the release of CDM, particularly that adsorbed in the nanomaterial, in the second releasing stage (10 days), whereas the steady releasing stage of CHG120 soaked in the same medium covered a shorter period of release (only 7 days) (Figure 5a). The double-matrix structure of FCHG120, consisting of the well-designed hydrogel structure and drug-carrier composition, enabled the more controlled and sustained release of CDM in the surrounding interstitial fluid-like medium (SIF). Consequently, CDM was liberated daily at higher concentrations from FCHG120, rather than CHG120 (Table S4), with an unperturbed duration of the burst releasing stage, but a longer period of the slow releasing stage.

**Figure 6:**
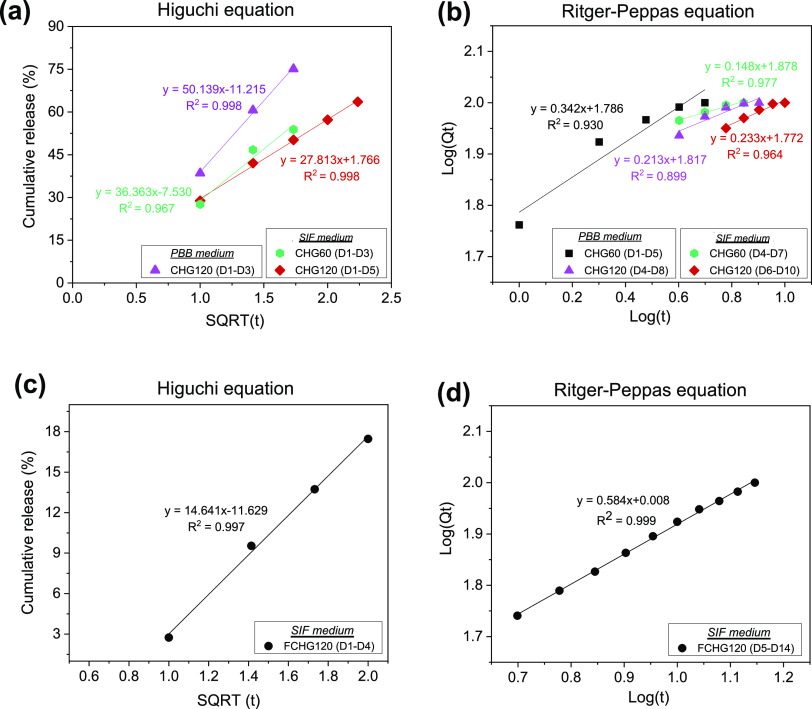
Release kinetics of GGOH from as-produced hydrogels (a, b) and FCHG120 (c, d) after being incubated in PBB and SIF for 14 days. The linear regression plots in Phase I and Phase II were fitted by the Higuchi and Ritger–Peppas equations, respectively.

Figure 6 shows the biphasic drug release patterns of GGOH from the porous hydrogels. The linear regression plots were created from the obtained drug-releasing profiles and fitted by the Higuchi and Ritger–Peppas equations in Phase I and II releases with the greatest values of the regression coefficient (*R*^2^) in the ranges of 0.967–0.998 and 0.899–0.999, respectively (Tables S6 and S7). The release mechanism, described by the Higuchi correlation, was derived using the pseudo-steady-state assumption, which assumes that the drug delivery system is controlled by the following criteria: the fraction of the drug released is proportional to the square root of releasing time; the initial drug concentration (total amount of drug loaded) is much higher than the drug solubility in an oil-in-water system, and swelling or dissolution of the carrier is negligible.^34^ The Higuchi rate constant (*K*_H_), which was determined from the slope of a plot, was directly correlated with the drug diffusivity. In Figure 6a, the *K*_H_ value calculated from the GGOH release profile of CHG120 soaked in PBB was almost double that determined from the hydrogel incubated in SIF, indicating that the GGOH diffusion occurred more readily in PBB than SIF. The presence of hydrophobic proteins in SIF markedly slowed down the diffusion of GGOH from the hydrogel matrix but helped lengthen the release of GGOH absorbed primarily in the CM matrix of CHG120. The diffusivity of GGOH from both CHG60 and CHG120 incubated in SIF appeared insignificantly different, but when the hydrogels were incubated in PBB, which was less hydrophobic than SIF, the early release stage of GGOH from CHG60 turned out to be less manipulated by the diffusivity of GGOH. As revealed in (Table S5), being loaded with a lower amount of drug, CHG60 demonstrated a much shorter prolonged liberation of GGOH (only 5 days), unlike CHG120. Such a short release duration could not be separately fitted to two equations. Hence, the release kinetic of GGOH in this hydrogel was solely described by the Ritger–Peppas correlation (Figure 6b). Overall, the sustained releases (Phase II) of CHG60 (in SIF) and CHG120 (in both media) were extended up to 5 days with the calculated *n* values in the range of 0.148–0.233, indicating a non-typical Fickian diffusion with some physicochemical interferences influencing the release, e.g., the diffusion of the less hydrophobic drug (GGOH) in the hydrophilic hydrogel matrix(CM).

Phase I release kinetic of GGOH from FCHG120 soaked in SIF was also expressed by the Higuchi model (Figure 6c). A relatively lower diffusivity (*K*_H_) of GGOH was attained in FCHG120, compared to that observed in CHG120. Most of the GGOH that were released in the first 4 days from FCHG120 by the diffusion-controlled release system was the drug freely located in the fibrin gel, not that forming the intermolecular interaction with fibrinogen in the fibrinogen/thrombin/CaCl_2_ solution during the preparation of FCHG120. The Phase II steady-state release of GGOH from FCHG120 was described by the Ritger–Peppas correlation (Figure 6d). The calculated *n* value was rather high at 0.584, indicating that the release mechanism of GGOH in this later phase was governed by both diffusion and swelling or erosion (anomalous transport).^35,36^ The presence of fibrin gel inside the CHG120 pores and surrounding outside the CHG hydrogel not only sustained the release of GGOH in the second phase up to at least 10 days but allowed the drug to be liberated daily at a fairly steady concentration via the gradual degradation of fibrin fibers by proteins in SIF.

### 3.4 In Vitro Antibacterial Activity of CG-Loaded Porous Hydrogels

**Figure 7:**
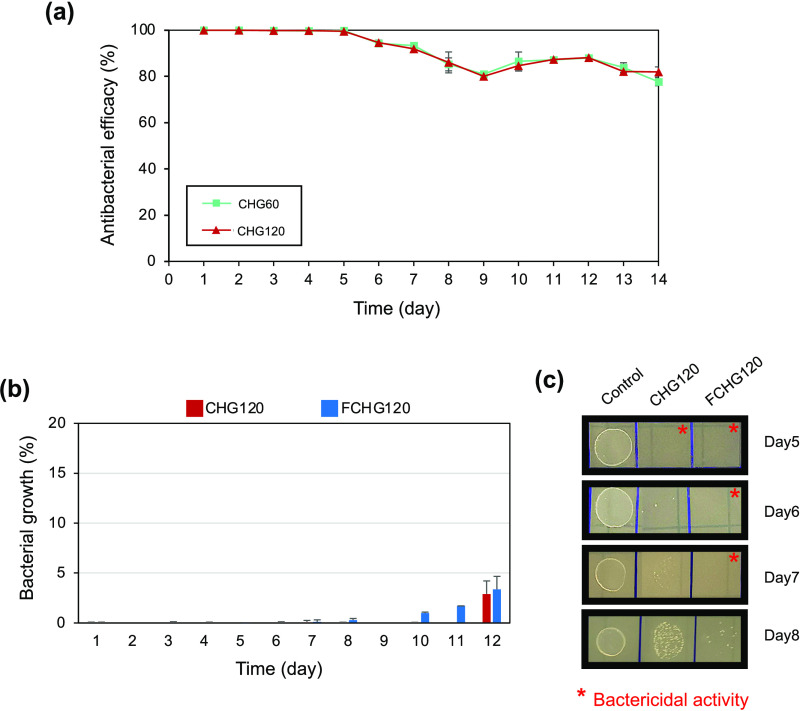
Antibacterial activity of the CG-loaded porous hydrogels against *Streptococcus sanguinis*: antibacterial efficacy (%) of CHG60 and CHG120 during exposure to the bacteria for 14 days (a), bacterial growth (%) of CHG120 and FCHG120 during exposure to the bacteria for 12 days (b), and a representative drop plate testing showing bacterial colonies (c), which was obtained from Day 5 to Day 8 bacterial supernatants of the experiments from (b). The data are presented as the mean percentage ± SD from three independent experiments.

The antibacterial activity of CHG60 and CHG120 against *Streptococcus sanguinis* is summarized in Figure 7a, which shows that the antibacterial efficiencies of both hydrogels were high at about 99–100% for the first 5 days and slightly decreased to approximately 90% on Day 7 and 80–90% between Day 7 and Day 14. The presence of a superior dose of GGOH in the CDM-loaded hydrogel seemed to impede the antibacterial potency of the composite hydrogel. When CHG120 was encapsulated by the fibrin matrix (mimicking the very early phase of a wound), the resulting material, i.e., FCHG120, as well as the starting CHG120, almost completely inhibited the bacterial growth for 8–9 days, as observed by the assessment of OD600 (Figure 7b). However, the bactericidal effect of CHG120 was observed only for the first 5 days, whereas that of FCHG120 was sustained until Day 7 (Figure 7c). The prolonged bactericidal activity of FCHG120 corresponded to the relatively more controlled release of CDM from the material. FCHG120 liberated higher CDM concentrations on Day 6 and Day 7, compared with those freed from CHG120 (Table S4).

*Streptococcus sanguinis*, a member of the viridans streptococcus group typically identified in BRONJ,^3,5^ was susceptible to CDM released from the CG-loaded composite hydrogels. The minimum bactericidal concentration (MBC) and the minimum inhibitory concentration 90 (MIC_90_) of *Streptococcus sanguinis* were 0.1 and 0.05 μg/mL, respectively (data not shown). Although the levels of CDM liberated from both CHG120 and FCHG120, quantified by HPLC analysis, appeared higher than the MBC value throughout the duration investigated, their bactericidal effects could be observed in the first 5 and 8 days, respectively. These could be possibly associated with the different kinetics of drug release resulted from the usage of two different media, i.e., SIF and broth culture with bacteria. It was previously reported that oral streptococci could activate plasminogen, leading to the degradation of fibrin,^37^ and express *N*-acetyl-β-d-glucosaminidase^38^ that may degrade *N*-acetyl-d-glucosamine in carboxymethyl chitosan. Upon the degradation of the polymer matrix (CM) of CHG120 and the fibrin matrix of FCHG120, CDM was more relatively quickly released into a bacterial culture medium, resulting in diminished amounts of drug liberated during the prolonged incubation period. This explained why the antibacterial activity of the CG-loaded composite hydrogels submerged in the broth culture with *Streptococcus sanguinis* did not last as long as expected from the HPLC results. Interestingly, during a longer incubation period (Day 10 to Day 12), FCHG120 lost its antibacterial activity more promptly than CHG120 (Figure 7b) despite its more prolonged release of CDM in SIF (Table S4), strongly confirming that the fibrin matrix had been gradually degraded, and hence, CDM absorbed in the degraded fibrin gel was simultaneously lost.

### 3.5 Reversal Activity of ZA Cytotoxicity by CG-Loaded Porous Hydrogels in MSCs

**Figure 8:**
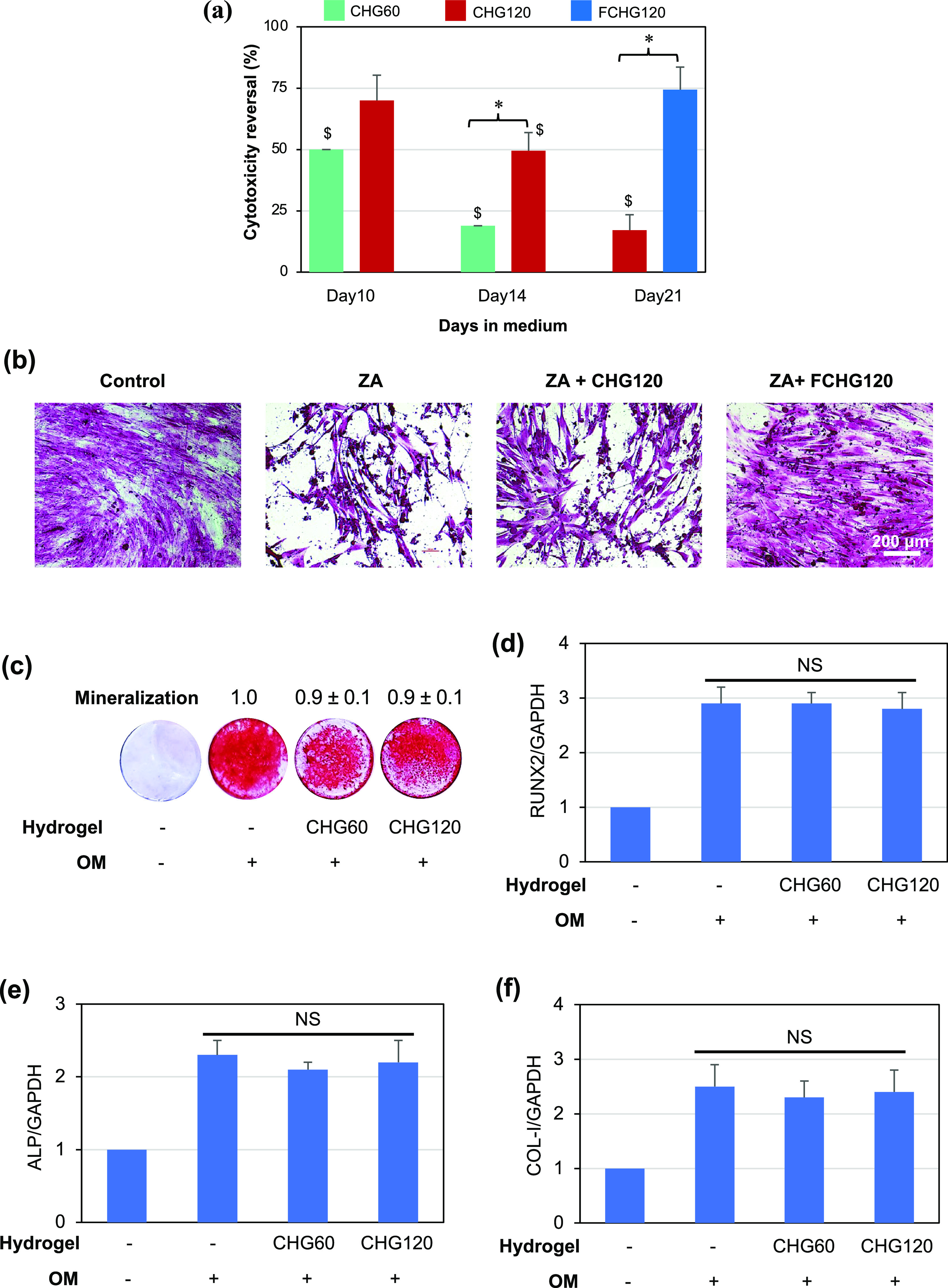
ZA cytotoxicity reversal activity of CHG60, CHG120, and FCHG120 on MSCs. MSCs were cultivated in a standard culture medium supplemented with ZA (5 μM) in the presence or absence of CG-loaded porous hydrogels, and the reversal activity against ZA cytotoxicity and cell morphology were determined by MTT (a) and crystal violet staining (b) on given days. In the absence of ZA and CG-loaded hydrogel, surviving MSCs were then further induced in OM for 21 days (for a mineralization assay) or 7 days (for a gene expression assay). Mineralization was examined by alizarin red S staining (c), and the expressions of RUNX2 (d), ALP (e), and COL-I (f) genes were measured by qPCR. The ZA cytotoxicity reversal data are presented as the mean percent ± SD from three independent experiments. ^$^*p* < 0.05 vs untreated cells (100%). The alizarin red S-stained samples shown are representative of three separate experiments, and the numbers indicate the mean fold-change ± SD, defined as 1.0 in the OM-induced naïve cells. The mRNA expression data are presented as the mean fold-change ± SD from three independent experiments, defined as 1.0 in the OM-induced naïve cells. *p < 0.05; NS: no significance.

The ZA cytotoxicity reversal (%) was calculated using eq 9 shown in Section 2.9.1 where MSCs cultured in the absence of ZA and any CG-loaded hydrogel specimen for 10 and 14 days were used as Day 10 and Day 14 controls for the CHG60 vs CHG120 experiments, respectively, while the Day 21 control for the FCHG120 experiment was those cultured in the absence of ZA and any CG-loaded hydrogel sample for 7 days. As revealed in Figure 8a, both CHG60 and CHG120 satisfactorily reversed the cytotoxicity of ZA by more than 50% for up to 10 days in culture. Their ZA toxicity reversal activity became less pronounced on Day 14, which was likely attributed to the lower level of GGOH released from the CG-loaded composite hydrogels. The reversal ability against ZA cytotoxicity of CHG120 was, however, significantly higher than that of CHG60 (50% vs 20%). The effect of the fibrin matrix in FCHG120 on the ability to reverse ZA toxicity was determined in comparison with that of CHG120 after both hydrogels were individually incubated in SIF for 14 days and subsequently brought into the cell culture insert used in the culture of ZA-treated MSCs for 7 days, corresponding to a 21-day release of GGOH. On Day 21, FCHG120 showed a significantly greater cytotoxicity reversal than CHG120 (75% vs less than 20%). Moreover, the morphological analysis results in Figure 8b revealed a discernible suppressive effect of a 7-day ZA treatment on the number of viable MSCs. Cells co-treated with ZA and each of CG-loaded porous hydrogels, which had previously been immersed in a SIF-releasing medium for 14 days, showed entirely different MCS morphologies. The healthier morphology of MSCs, almost comparable to that of the cells without ZA treatment (used as a control), was explicitly observed when FCHG120 was in use, indicating that the hydrogel successfully protected MSCs from ZA toxicity (Figure 8b). The effective cytoprotective concentrations of GGOH for MSCs against ZA were previously reported to be 5–75 μM.^6^ It is thus possible that FCHG120 sustained the release of GGOH, for at least 14 days in SIF plus 7 days in a culture medium, at sufficient levels against ZA during the 7-day MSC culture. The results suggested the biological importance of fibrin as a natural barrier that favorably controlled the release of drugs from CHG120.

The osteogenic function and differentiation of MSCs that survived ZA toxicity were investigated, and the results are shown in Figure 8c–f. In the presence of osteogenic induction, surviving MSCs produced biomineralization comparable to that formed by MSCs naïve to ZA (Figure 8c). The ability of surviving MSCs to express RUNX2, ALP, and COL-I, under osteogenic stimulation, was also comparable to that of MSCs naïve to ZA (Figure 8d–f, respectively). The results indicated that both CHG60 and CHG120 rescued MSCs from ZA cytotoxicity despite their different ZA reversal potentials. These differences were attributed to the varied amounts of GGOH initially loaded and its release behaviors. Very few viable MSCs were produced by ZA pre-treatment without CG-loaded composite hydrogel, and these MSCs did not recover upon subsequent culture (data not shown). Thus, assays could not be performed on this group.

### 3.6 Increased In Vitro Cytocompatibility and In Ovo Biocompatibility by Fibrin Matrix in FCHG120

**Figure 9:**
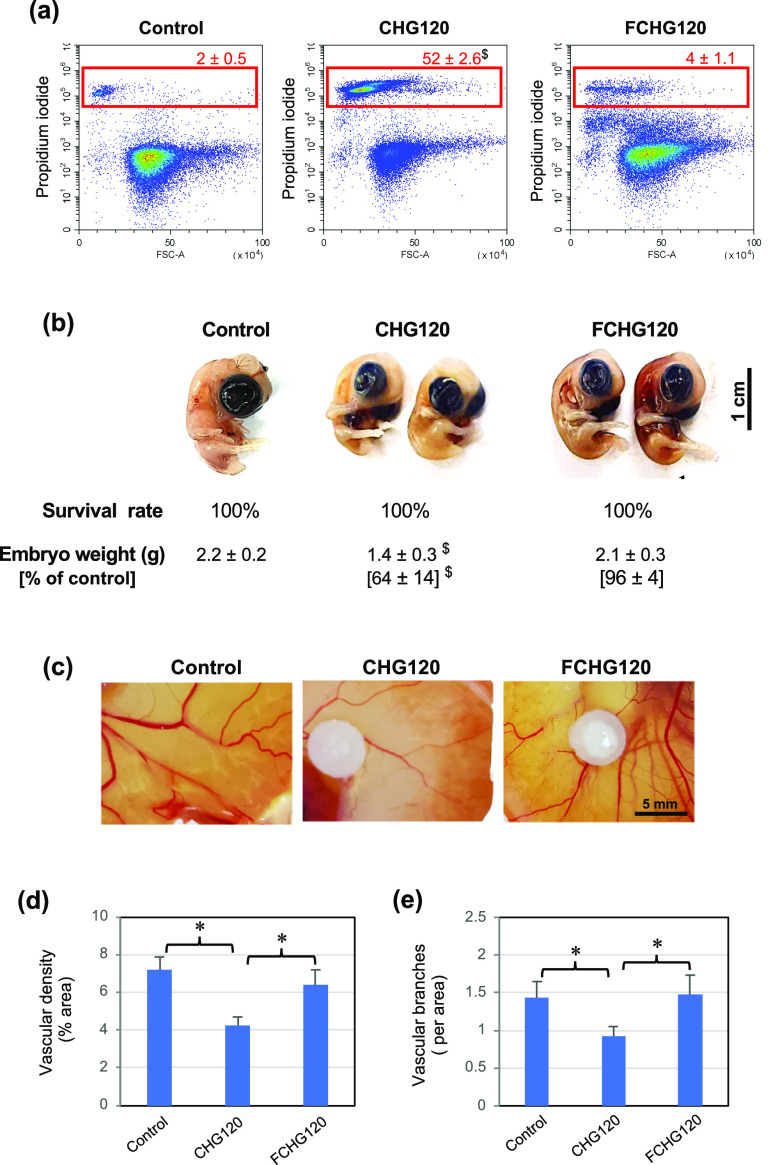
In vitro and in ovo biocompatibilities of CHG120 and FCHG120. The in vitro cytocompatibility to monocyte-like cells (a) and the in ovo acute toxicity on tissue formation (b) and angiogenesis (c–e) using a CAM model. The data are presented as the mean percent ± SD from three independent experiments. ^$^*p* < 0.05 vs control sample. *p < 0.05.

The cytocompatibility of freshly prepared CHG120 and FCHG120 without a 24 h pre-incubation with a culture medium was assessed using monocyte-like RAW cells, which represent an important acute inflammatory cell type crucial for the early healing process. The results in Figure 9a reveal that while CHG120 caused 52% propidium iodide-positive cell death, FCHG120 reduced cell death to only 4%, comparable to that in the control cells. During the first 48 h in culture, the estimated accumulative concentrations of CDM and GGOH released from CHG120 were approximately 200 μg/mL and 160 μM, respectively, while those of FCHG120 were about 80 μg/mL and 35 μM, respectively (Tables S4 and S5, respectively). The markedly lesser burst releases of both drugs from FCHG120 appeared to increase cytocompatibility to monocyte-like RAW cells. The monocyte/macrophage lineage cells have been shown to play an important role during multiple overlapping phases of bone healing.^39^ A chick egg model was also employed to assess the in ovo biocompatibility of the CG-loaded porous hydrogels, as shown in (Figure 9b), both CHG120 and FCHG120 did not cause embryo lethal. However, CHG120 retarded the growth of embryos weighing only 64% of the control. In contrast, growth retardation was not observed in the FCHG120 group. Moreover, the CAM treated with CHG120 was less vascularized, compared with that of the control (untreated CAM) (Figure 9c). Analysis of the CAM blood vessels in Figure 9d,e indicated that CHG120 significantly decreased vascular density and the number of blood vessel branches. Both in vitro and in ovo data vividly demonstrated that FCHG120 possessed significantly higher biocompatibility than CHG120 and appeared non-toxic (comparable to the untreated control group).

Though, in this study, the simulated fibrin matrix significantly modulated the suppressive effect of CHG120 on the viability of monocyte/macrophage cells and chick embryonic tissue growth and angiogenesis, resulting in a highly biocompatible FCHG120. However, it is important to note that in the in vivo implantation of a material, the body quickly responds within minutes by forming a fibrin matrix surrounding the material, subsequently increasing multilayers of fibrin along with many other plasma proteins directly attached to the material surface. These include fragmented structural biomolecules (such as elastin, collagen, and fibrin/fibrinogen), tryptophan-, tyrosine- and phenylalanine-containing proteins, and certain coenzymes (such as flavins and pyridine nucleotides).^28,40,41^ The actual modulatory role of the fibrin matrix in controlling the interaction between the CG-loaded composite hydrogels and biologics in the microenvironment at the implantation site remains unclear and needs to be studied further. Momentarily, it is important to note that the composite hydrogel of highly interconnected porous CM and plasma-treated nano-sized mesoporous silica nanoparticles, well-regulated the delivery of dual-functional drugs, i.e., CDM and GGOH, enabling CHG120 to elicit antibacterial and ZA cytotoxicity reversal activities. It helped preserve the MSC viability and a pool of viable MSCs, that undergo normal osteogenic differentiation and produce mineralization. These dual activities of CHG120 were sustained for a duration that is crucial to protect the healing of extraction socket wound from oral bacterial infection before complete coverage epithelialization,^42^ as well as mediate the reversal of ZA cytotoxicity during the healing process.^42,43^

## 4 Conclusions

A dual-functional drug delivery system for the prevention of MRONJ associated with ZA was successfully developed via the preloading of water-soluble CDM in PMCM-41 prior to the fabrication and steam-induced crosslinking of lyophilized CDM-loaded PMCM-41-integrated carboxymethyl chitosan pads, followed by the post-loading of GGOH onto the hydrogel. The nano-sized mesoporous silica particles incorporated in the hydrogels (CHG), as well as the differences in hydrophilicity of the hydrogel matrix, the drugs loaded, and the releasing media, enabled the prolonged releases of both CDM and GGOH with releasing quantities of each drug sufficient to exhibit bactericidal activity against *Streptococcus sanguinis* for at least 5 days and reverse the ZA cytotoxicity to MSCs by more than 50% for 14 days in culture, respectively. In a more physiological-like in vitro model, the fibrin network in FCHG not only further markedly subsided the burst releases but also considerably extended the sustained releases of the drugs loaded with some changes in the release kinetics but still most perfectly fitted by the Ritger–Peppas and Higuchi models. Consequently, the bactericidal and ZA cytotoxicity reversal activities of FCHG were more sustained over 7 and 21 days in culture, respectively. Furthermore, the in vitro and in ovo acute drug toxicity of CHG could be reasonably lessened when encompassed with the fibrin clot. This would positively warrant further in vivo studies.

## Supporting Information Available

Additional experimental details; reagents used for the preparation of simulated body fluid (SBF) solution; schematic diagram of the cell culture with the dual drug-loaded composite hydrogel; experimental conditions of HPLC analysis of clindamycin (CDM) and geranylgeraniol (GGOH); primer sequences used in the study; concentrations of CDM released from the dual drug-loaded porous hydrogels; concentrations of GGOH released from the dual drug-loaded porous hydrogels; and linear regression coefficients (*R*^2^) values (PDF)
