# Supplementary material for: Dual-Functional Drug Delivery System for Bisphosphonate-Related Osteonecrosis Prevention and Its Bioinspired Releasing Model and In Vitro Assessment
Source: ACS Omega. 2023 Jul 14;8(29):26561–76. doi: 10.1021/acsomega.3c03440 (PMC10373185; doi:10.1021/acsomega.3c03440)
Supplement: Supplementary file 1 — ao3c03440_si_001.pdf [file ao3c03440_si_001.pdf]

Supporting Information

Additional experimental details and supporting data related to the Results Section

Dual-Functional Drug Delivery System for Bisphosphonate-Related Osteonecrosis Prevention and Its Bioinspired Releasing Model and In Vitro Assessment

Piyarat Sungkhaphan,[†,#] Boonlom Thavornyutikarn,[†,#] Papon Muangsanit,[‡] Pakkanun Kaewkong,[†] Setthawut Kitpakornsanti,[§] Soraya Pornsuwan,[‖] Weerachai Singhatanadgit[*,§] Wanida Janvikul[*,†]

[†]National Metal and Materials Technology Center, National Science and Technology Development Agency, Khlong Luang 12120, Thailand

[‡]National Center for Genetic Engineering and Biotechnology, National Science and Technology Development Agency, Khlong Luang 12120, Thailand

[§]Faculty of Dentistry and Research Unit in Mineralized Tissue Reconstruction, Thammasat University (Rangsit Campus), Khlong Luang 12120, Thailand

[‖]Faculty of Science, Mahidol University, Bangkok 10400, Thailand

*Email: s-wrch@staff.tu.ac.th.

*Email: wanidaj@mtec.or.th.

Additional experimental details

1. Detailed preparation of simulated body fluid (SBF)

Simulated body fluid (SBF) was prepared according to the Kokubo's method (1). Reagents (Table S1) were purchased from Sigma-Aldrich and used as received without further purification. To prepare 1000 mL of SBF, 700 mL of ultrapure water (Milli-Q water, 18.2 MΩ) was placed in a 1 L beaker and set at 37ºC ± 1.0ºC in a water bath. The reagents of the first to eighth orders were slowly added to the water one by one with an accuracy of ± 0.5 mg. The solution was continuously stirred. The pH was monitored to avoid a rapid increase, resulting in precipitation. The reagent of the ninth order (Tris) was dissolved in ultrapure water (30 mL) before being added into the mixture. After mixing all reagents, the pH of the resulting mixture was adjusted to 7.4 using a small amount of 1M HCl. Then, the SBF solution was transferred to a 1 L volumetric flask and filled to the mark with ultrapure water after cooling at room temperature. The resulting SBF solution was colorless and transparent.

Table S1. Reagents used for the preparation of simulated body fluid (SBF) solution

Order	Reagent	Amount (g/L)
1	Sodium chloride (NaCl)	8.035
2	Sodium hydrogen carbonate ($NaHCO_3$)	0.355
3	Potassium chloride (KCl)	0.225
4	Potassium hydrogen phosphate trihydrate ($K_2HPO_4.3H_2O$)	0.231
5	Magnesium chloride hexahydrate ($MgCl_2.6H_2O$)	0.311
6	1.0 M hydrochloric acid (HCl)	39 mL
7	Calcium chloride ($CaCl_2$)	0.292
8	Sodium sulfate (Na_2SO_4)	0.072
9	Tris (Hydroxymethyl aminomethane)	6.118

2. Detailed preparation of simulated interstitial fluid (SIF)

In the present study, SBF containing 10% human serum (SIF) was used as a releasing medium mimicking interstitial fluid. Interstitial fluid has ionic concentrations nearly similar to human plasma and much lower protein concentration (2). Human serum was prepared by defibrination of an anticoagulant buffy coat pooled plasma with the addition of calcium chloride (25 mM) at 4ºC overnight, followed by refrigerated centrifugation at 3000 × g for 30 min. The use and handling of the buffy coat blood product, kindly provided by Thammasat University Hospital Blood Bank, were approved by the Ethics Review Sub-Committee for Research Involving Human Research Subjects of Thammasat University No. 3 (049/2564) and the Institutional Biosafety Committee Thammasat University (034/2564). Permission for the use of buffy coats was obtained from Thammasat University Hospital Blood Bank.

Table S2. Experimental conditions of HPLC analysis of clindamycin (CDM) and geranylgeraniol (GGOH)

Condition	CDM	GGOH
Column	ODV-100V, C18, 4.6 mm × 150 mm, 5 µm particle size	
Mobile phase	70 : 30 (v/v) mixed solvent of 0.02 M disodium hydrogen phosphate (Na_2HPO_4) (pH = 2.5) and acetonitrile	90 : 10 (v/v) mixed solvent of acetonitrile and double deionized water
Flow rate	0.5 mL/min	0.5 mL/min
Injection volume	100 µL	100 µL
Detection wavelength	210 nm	200 nm

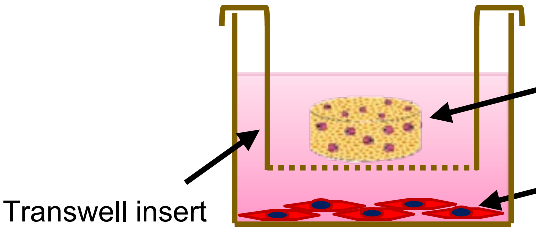

Figure S1. A schematic diagram of the cell culture with the dual drug-loaded composite hydrogel.

Table S3. Primer sequences used in the study

Gene	Forward sequence	Reverse sequence
Human RUNX2	5′-TGGTTACTGTCATGGCGGGTA-3′	5′-TCTCAGATCGTTGAACCTTGCTA-3′
Human ALP	5′-ACTGGTACTCAGACAACGAGAT-3′	5′-ACGTCAATGTCCCTGATGTTATG-3′
Human COL-I	5′-GAGGGCCAAGACGAAGACATC-3′	5′-CAGATCACGTCATCGCACAAC-3′
Human GAPDH	5′- CTGGGCTACACTGAGCACC-3′	5′- AAGTGGTCGTTGAGGGCAATG-3′

Supporting data related to the Results Section

Table S4. The concentrations of CDM released from the dual drug-loaded porous hydrogels incubated in different releasing media, i.e., PBB and SIF, for 14 days, measured by HPLC analysis*

Day	CDM concentration (µg/mL)				
	CHG60		CHG120		FCHG120 (SIF)
	PBB	SIF	PBB	SIF	
1	196.1 ± 22.5	154.4 ± 11.4	204.7 ± 26.4	145.4 ± 18.1	57.2 ± 7.9
2	16.4 ± 1.6	11.7 ± 2.2	19.1 ± 0.9	14.2 ± 3.5	21.2 ± 1.3
3	3.3 ± 0.2	7.3 ± 0.1	3.1 ± 0.1	5.0 ± 0.9	7.2 ± 1.0
4	2.0 ± 0.1	3.2 ± 0.1	1.5 ± 0.1	5.5 ± 1.1	7.3 ± 0.2
5	1.7 ± 0.1	2.9 ± 0.2	1.1 ± 0.1	1.5 ± 0.3	2.7 ± 0.1
6	1.4 ± 0.1	2.0 ±0.2	0.9 ± 0.1	1.6 ± 0.2	2.7 ± 0.4
7	1.4 ± 0.1	1.0 ± 0.2	0.8 ± 0.1	1.7 ± 0.0	2.0 ± 0.1
8	1.0 ± 0.1	0.6 ± 0.0	0.3 ± 0.1	0.8 ± 0.1	3.0 ± 0.1
9	0.8 ± 0.1	0.6 ± 0.3	0	1.1 ± 0.1	2.0 ± 0.1
10	0.8 ± 0.1	0.4 ± 0.1	0	1.2 ± 0.1	1.5 ± 0.1
11	0	0.4 ± 0.0	0	0.8 ± 0.2	1.5 ± 0.1
12	0	0.4 ± 0.2	0	0	1.1 ± 0.1
13	0	0	0	0	0.8 ± 0.1
14	0	0	0	0	0.8 ± 0.1
Total (µg)	269.7 ± 27.9	222.0 ± 17.5	256.8 ± 15.7	214.5 ± 15.6	132.9 ± 13.3

* PBB was a phosphate-buffered saline (PBS)-based mixed fluid containing PBS mixed with 10% fetal bovine serum, while SIF was the fluid containing SBF mixed with 10% human serum.

Table S5. The concentrations of GGOH released from the dual drug-loaded porous hydrogels incubated in different releasing media, i.e., PBB and SIF, for 14 days, measured by HPLC analysis

**Day**	**GGOH concentration (μM)**				
	CHG60		CHG120		FCHG120 (SIF)
	PBB	SIF	PBB	SIF	
1	77.2±0.3	47.5 ± 0.6	132.6±4.0	112.0 ± 8.8	9.4 ± 3.5
2	34.8±2.7	32.9 ± 2.9	76.1±8.4	48.4 ± 0.8	23.4 ± 2.1
3	11.5±0.3	12.3 ± 1.0	49.9±2.8	26.7 ± 1.5	14.4 ± 2.8
4	7.1±1.7	8.2 ± 0.4	34.0±0.5	25.3 ± 1.5	12.9 ± 3.7
5	2.7±0.4	3.9 ± 0.3	26.1±1.8	22.3 ± 0.8	10.2 ± 2.3
6	0	3.4 ± 0.1	13.1±3.0	19.2 ± 0.3	8.4 ± 0.9
7	0	1.1 ± 0.1	6.1±1.3	12.7 ± 0.2	7.0 ± 1.2
8	0	0	1.2±0.2	12.2 ± 1.3	7.5 ± 2.1
9	0	0	0	6.3 ± 0.9	7.2 ± 1.4
10	0	0	0	1.9 ± 0.1	6.8 ± 2.0
11	0	0	0	0	6.2 ± 1.1
12	0	0	0	0	4.3 ± 0.9
13	0	0	0	0	5.1 ± 0.3
14	0	0	0	0	5.1 ± 0.3
Total (μg)	45.9 ± 1.5	38.1 ± 1.6	118.2 ± 1.8	100.1 ± 1.8	44.5 ± 6.9

Table S6. Linear regression coefficients (R^2) values obtained after fitting individual mathematical models to the CDM or GGOH release profiles of the CG-loaded porous hydrogels soaked in PBB for 14 days

**Sample**	**First order**		**Higuchi**		**Weibull**		**Ritger-Peppas**		**Hixson Crowell**	
**CDM**	Rapid stage	Steady stage	Rapid stage	Steady stage	Rapid stage	Steady stage	Rapid stage	Steady stage	Rapid stage	Steady stage
CHG60	0.874	0.953	0.923	0.977	0.955	0.991	0.956	0.991	0.875	0.953
CHG120	0.848	0.963	0.901	0.979	0.938	0.989	0.938	0.990	0.849	0.963
**GGOH**	Phase I	Phase II	Phase I	Phase II	Phase I	Phase II	Phase I	Phase II	Phase I	Phase II
CHG60	0.773		0.899		0.929		0.930		0.790	
CHG120	0.959	0.830	0.998	0.877	0.997	0.888	0.997	0.899	0.969	0.833

Table S7. Linear regression coefficients (R^2) values obtained after fitting individual mathematical models to the CDM or GGOH release profiles of the CG-loaded porous hydrogels soaked in SIF for 14 days

**Sample**	**First order**		**Higuchi**		**Weibull**		**Ritger-Peppas**		**Hixson Crowell**	
**CDM**	Rapid stage	Steady stage	Rapid stage	Steady stage	Rapid stage	Steady stage	Rapid stage	Steady stage	Rapid stage	Steady stage
CHG60	0.902	0.967	0.966	0.982	0.991	0.992	0.992	0.997	0.906	0.967
CHG120	0.918	0.984	0.967	0.995	0.988	0.984	0.988	0.992	0.922	0.985
FCHG120	0.882	0.953	0.962	0.981	0.751	0.987	0.974	0.992	0.895	0.954
**GGOH**	Phase I	Phase II	Phase I	Phase II	Phase I	Phase II	Phase I	Phase II	Phase I	Phase II
CHG60	0.901	0.949	0.967	0.968	0.917	0.977	0.912	0.977	0.912	0.951
CHG120	0.923	0.933	0.998	0.954	0.975	0.964	0.975	0.964	0.946	0.934
FCHG120	0.861	0.970	0.997	0.998	0.965	0.998	0.966	0.999	0.909	0.978

References

(1) Kokubo T, Takadama H. How useful is SBF in predicting in vivo bone bioactivity? Biomaterials. 2006;27(15):2907-15.

(2) Fogh-Andersen N, Altura BM, Altura BT, Siggaard-Andersen O. Composition of interstitial fluid. Clin Chem. 1995;41(10):1522-5.
